# Bifrontal electroconvulsive therapy leads to improvement of cerebral glucose hypometabolism in frontotemporal dementia with comorbid psychotic depression – a case report

**DOI:** 10.1186/s12888-023-04759-z

**Published:** 2023-04-20

**Authors:** Sebastian Schröder, Lena Bönig, Phileas Johannes Proskynitopoulos, Eva Janke, Johannes Heck, Nima Mahmoudi, Adrian Groh, Georg Berding, Felix Wedegärtner, Stephanie Deest-Gaubatz, Hannah Benedictine Maier, Stefan Bleich, Helge Frieling, Martin Schulze Westhoff

**Affiliations:** 1 Department of Psychiatry, Social Psychiatry and Psychotherapy, Hannover Medical School, Carl-Neuberg-Str. 1, 30625 Hannover, Germany; 2 Department of Neurology, Hannover Medical School, Hannover, Germany; 3 Institute for Clinical Pharmacology, Hannover Medical School, Hannover, Germany; 4 Department of Diagnostic and Interventional Neuroradiology, Hannover Medical School, Hannover, Germany; 5 Department of Nuclear Medicine, Hannover Medical School, Hannover, Germany

**Keywords:** ^18^F-FDG PET/CT, Frontotemporal dementia, ECT, Depression

## Abstract

**Background:**

Differentiating depression and dementia in elderly patients represents a major clinical challenge for psychiatrists. Pharmacological and non-pharmacological treatment options for both conditions are often used cautiously due to fear of adverse effects. If a clinically indicated therapy is not initiated due to fear of adverse effects, the quality of life of affected patients may significantly be reduced.

**Case presentation:**

Here, we describe the case of a 65-year-old woman who presented to the department of psychiatry of a university hospital with depressed mood, pronounced anxiety, and nihilistic thoughts. While several pharmacological treatments remained without clinical response, further behavioral observation in conjunction with ^18^F-fluoro‐2‐deoxy‐D‐glucose positron emission tomography/computed tomography (^18^F-FDG PET/CT) revealed the diagnosis of frontotemporal dementia (FTD). To counter the pharmacological treatment resistance of psychotic depression, we decided to perform electroconvulsive therapy (ECT). Remarkably, ten sessions of ECT yielded an almost complete remission of depressive symptoms. In addition, the patient’s delusional ideas disappeared. A follow-up ^18^F-FDG PET/CT after the ECT series still showed a frontally and parieto-temporally accentuated hypometabolism, albeit with a clear regression compared to the previous image. The follow-up ^18^F-FDG PET/CT thus corroborated the diagnosis of FTD, while on the other hand it demonstrated the success of ECT.

**Conclusions:**

In this case, ECT was a beneficial treatment option for depressive symptoms in FTD. Also, ^18^F-FDG PET/CT should be discussed as a valuable tool in differentiating depression and dementia and as an indicator of treatment response.

## Background

Frontotemporal dementia (FTD) comprises various clinical manifestations reflecting neurodegenerative processes in the frontal and temporal lobes [1]. Neuropathologically, FTD is driven by neuronal loss and microvacuolar changes in these brain regions, causing deficits in behavior, language, and executive functions [2]. Among the various clinical manifestations of FTD, the behavioral variant (bv-FTD) is the most widespread symptom complex, including stereotypical behavior, executive dysfunction, loss of empathy, and disinhibition resulting in socially undesirable actions [2]. A characteristic feature of bv-FTD is apathy, which leads to social withdrawal and a loss of interest in hobbies, work, or personal hygiene [2]. The bv-FTD is historically known as pick’s disease after the first describer Arnold Pick [3].

In bv-FTD, the frontotemporal atrophy on cranial magnetic resonance imaging (cMRI) is preceded by bilateral amygdala, striatal, and insular atrophy, which explains the numerous neuropsychiatric symptoms [4].

In general, the diagnosis of FTD is difficult to establish due to numerous psychiatric and neurological differential diagnoses and due to the lack of specific diagnostic tests/biomarkers [5]. Electroencephalographic studies can be used for differential diagnosis of FTD compared with other dementias but cannot be considered a sensitive diagnostic marker [6]. Imaging techniques represent valuable tools to differentiate between depression and dementia, as they can precisely depict neurodegenerative alterations [7]. In the current international FTD guidelines for the diagnosis of bv-FTD, frontal and/or temporal abnormalities on conventional magnetic resonance imaging (MRI), fluorine-18-fluorodeoxyglucose positron emission tomography (^18^F-FDG-PET), or single photon emission computed tomography (SPECT) are required for the diagnosis of “probable” bv-FTD [8]. Higher levels of p-tau and t-tau and lower levels of Aβ1–42 can be detected in the cerebrospinal fluid (CSF) in patients with Alzheimer’s disease (AD) than in patients with FTD, but p-tau, t-tau, and Aβ1–42 are still not suitable as sensitive diagnostic markers [9, 10].

Due to its clinical heterogeneity, bv-FTD can mimic a depressive episode, especially its initial stages [7]. The border between FTD and depression often becomes blurred, as depressive symptoms conversely may precede dementia and characteristic manifestations such as depressive pseudodementia often occur in elderly people [11]. While there is a large armamentarium of pharmacological and non-pharmacological treatment options for affective disorders, no curative treatment for dementia exists to date, despite extensive research efforts [11]. FTD is the second most frequent cause of early-onset dementia and leads to death within 5 to 10 years after diagnosis [1]. FTD thus poses a substantial health and economic issue [1]. So far, only selective serotonin reuptake inhibitors (SSRIs) have been demonstrated to improve behavioral symptoms in FTD [2]. Even though electroconvulsive therapy (ECT) constitutes an established treatment option for numerous psychiatric disorders and probably is the most effective tool for treating depression, its use for depressive symptoms in the context of dementia has not been fully elucidated. As of today, there are only some promising case reports and case series available which describe the use of ECT for the treatment of dementia-related aggressiveness and agitation [12, 13]. The aim of our study was to document the clinically successful use of ECT in a patient with FTD with an improvement of frontotemporal hypometabolism evidenced by FDG-PET/CT.

## Case presentation

A 65-year-old woman was admitted to the department of psychiatry of a large German university hospital with referral from her general practitioner. The patient had worked as a community worker for several years, until she was signed off a few years ago. She complained about severely depressed mood, rumination, and restlessness. During the initial clinical assessment, she also reported thoughts of guilt and a possible addiction to benzodiazepines. Furthermore, subjective concentration deficits, loss of appetite, fear of failure, and feelings of inadequacy became obvious. The patient claimed that she felt “internally dead“ and “not belonging to this world“, suggesting delusional nihilistic ideation. With regard to these delusional contents, the patient was incorrigible. In addition, pronounced psychomotor agitation became apparent during the examination. The patient and her sons (one of whom acted as the patient’s legal guardian) reported an extensive history of depressive episodes; the last episode had occurred approximately 20 years ago. The mother of the patient had been diagnosed with Alzheimer’s disease at the age of 64 years and had died at the age of 73 years. Further somatic diagnoses of the patient comprised arterial hypertension, tachycardic atrial fibrillation, and status post myocardial infarction four years ago. Except for the mother of the patient, no other family members had been afflicted with neurological or psychiatric diseases. Taken together, a depressive episode with psychotic symptoms was assumed and the patient was transferred to an open ward of the department of psychiatry.

Pharmacological antidepressive treatment with sertraline and mirtazapine was initiated and well tolerated. Before, a treatment trial with duloxetine and moclobemide had led to increased restlessness and was therefore discontinued. An additional antipsychotic medication—initially with quetiapine and subsequently with amisulpride—resulted in akathisia and drowsiness and was stopped later. Finally, a combination of sertraline and mirtazapine with the antipsychotic agent olanzapine led to a mild improvement of depressive symptoms, while agitation and restlessness declined under treatment with lorazepam. However, therapeutic success remained insufficient, particularly because nihilistic ideation persisted, and pessimistic rumination continued over the following months. Due to psychotic symptoms and therapy resistance to conventional antidepressive drugs, electroconvulsive therapy (ECT) was considered. The patient was clinging, contacted the physicians and the nursing staff several times per day and expressed her despair and fears. She frequently complained about somatic symptoms such as constipation or abdominal pain without corresponding somatic correlate. Medication attempts with lithium and trazodone remained without substantial clinical effect. In addition, cognitive deficits frequently became apparent. Hence, we suspected an organic cause of the neuropsychiatric symptom complex.

Laboratory investigations revealed a mild chronic renal failure. Besides, hyponatremia was identified, and sodium chloride tablets were administered. No abnormalities were detected in the complete blood count. Deficiencies of folic acid, vitamin B_12_, and ceruloplasmin as well as infection with human immunodeficiency virus, *Borrelia burgdorferi*, and *Treponema pallidum* could also be excluded.

Further neurological examination neither revealed signs of Parkinsonism nor motoric nor sensory deficits. Solely concentration, attention, and short-term memory appeared to be reduced. Global cognitive function was assessed with the aid of the Montreal Cognitive Assessment (MoCA), in which the patient scored 15 out of 30 points, indicating moderate to severe cognitive deficits [14]. In addition, the Consortium to Establish a Registry on Alzheimer’s Disease neuropsychological assessment battery (CERAD-Plus) was utilized, which detected an impairment in several sections, especially in verbal recognition and visuospatial abilities [15]. By contrast, the frontal assessment battery (FAB), where the patient scored 12 out of 18 points, was inconspicuous [16]. The Inventory of Clinical Personality Accentuations (ICP) displayed unspecific results with regard to a possible personality disorder, while 61 out of 63 points in the Beck Depression Inventory II (BDI-II) demonstrated a severe depressive symptomatology [17, 18]. ICP and BDI-II were performed as self-questionnaires, whereas MoCA, CERAD-Plus, and FAB were conducted by a healthcare professional.

Although FAB had remained inconspicuous, we suspected bv-FTD due to repetitive stereotypic behavior and social disinhibition with accompanying depressive-like symptoms.

**Fig. 1:**
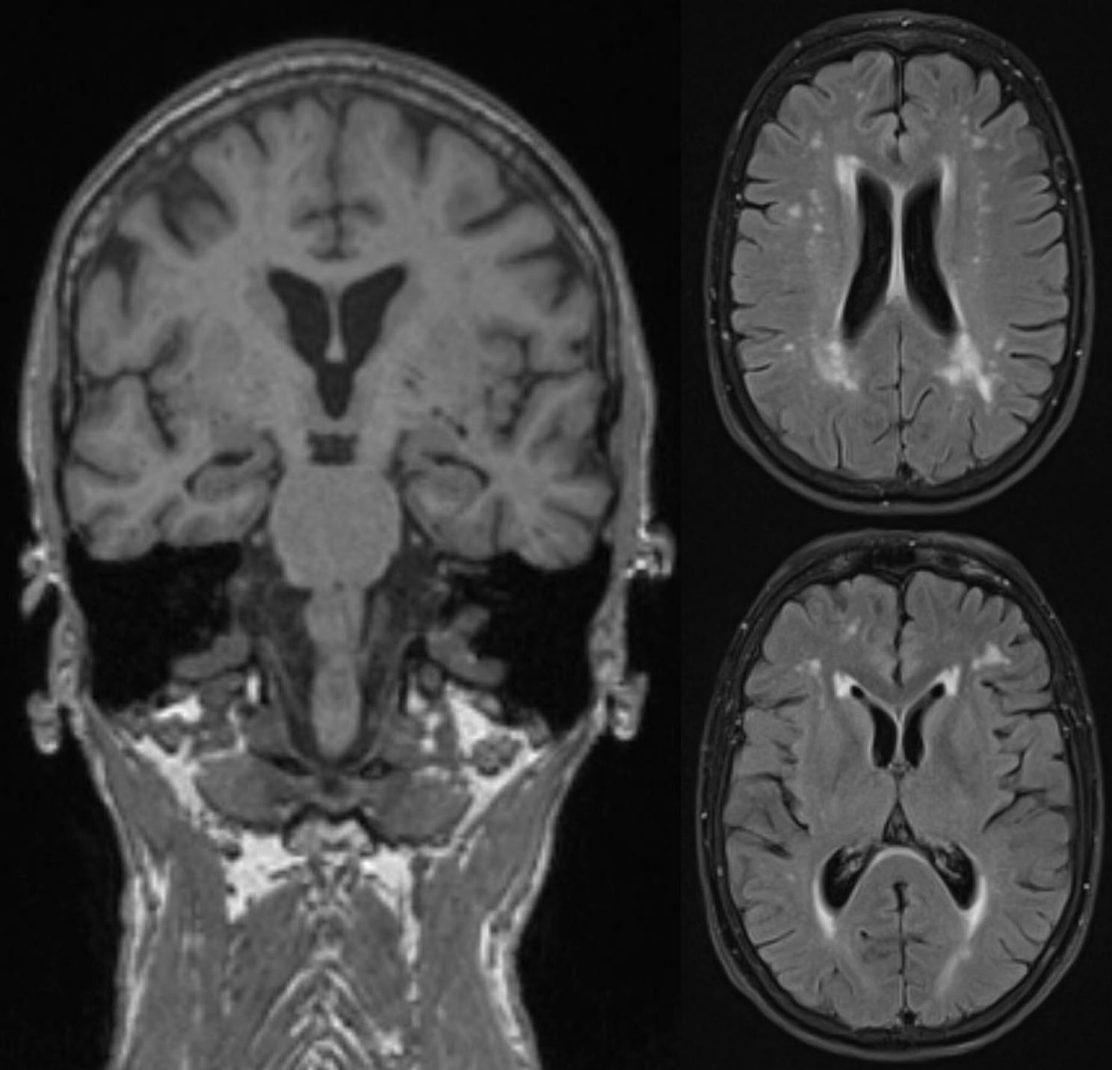
Magnetic resonance imaging (MRI) displaying bihemispheric, supratentorial T2 white matter hyperintensities

**Fig. 2:**
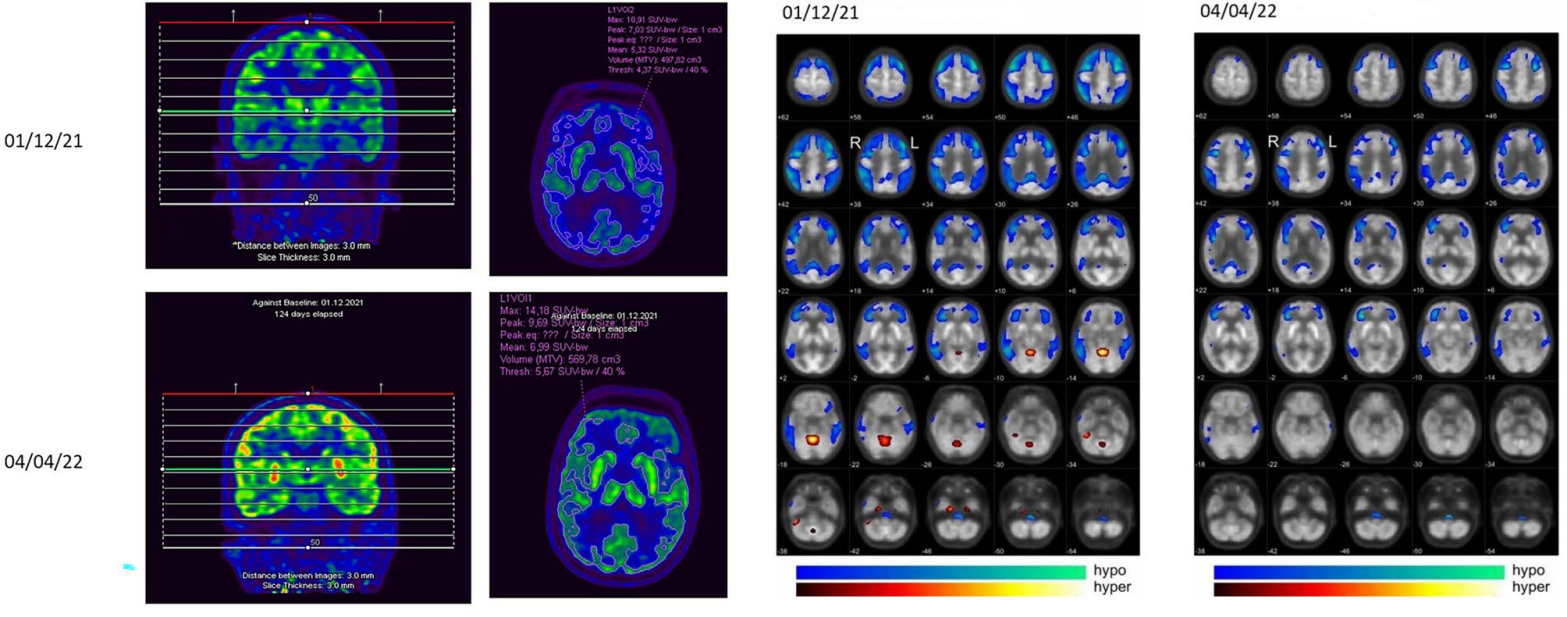
^18^F-FDG PET/CT depicting improvement of frontally accentuated glucose hypometabolism after treatment with ECT. Figure 2A indicates that the Standardized Uptake Value (SUV) peak in the CNS before therapy was 7.0, afterwards 9.7. In the tomograms scaled to the maximum of the follow-up study, the lower uptake before therapy is visible in several brain regions (cerebral cortex, basal ganglia, cerebellum). Figure 2B maps the Statistical Parametric Mapping (SPM) comparison of the respective studies to a control population. It shows before therapy a significantly more extended, contiguous cluster of suprathreshold voxels (p < 0.001) with significant hypometabolism, whereas after therapy four non-contiguous, significantly smaller clusters could be found

To expand the diagnostics, an electroencephalogram was conducted, which demonstrated a physiological alpha rhythm without signs of epileptiform potentials. Magnetic resonance imaging (MRI) revealed bihemispheric, supratentorial T2 white matter hyperintensities depicting moderate leukoencephalopathy according to Fazekas grade 2 (Fig. 1). A lumbar puncture with examination of CSF parameters including tau and beta-amyloid protein and its ratios was unremarkable. Moreover, intrathecal immunoglobulin synthesis and the presence of autoimmune encephalitis antibodies and protein 14–3–3 in CSF were excluded. In summary, we assumed a neurodegenerative process as the cause of the patient’s symptoms and initiated an examination of cerebral glucose metabolism by ^18^F‐FDG PET/CT. Herein, glucose uptake was bilaterally reduced in the frontal, temporal, and parietal cortex, while it was relatively higher in sensomotoric and occipital regions (Figs. 2A-B). This specific pattern of glucose hypometabolism clearly pointed to neurodegeneration in the sense of FTD. Therefore, in synopsis of all findings and symptoms, the diagnosis FTD was established according to ICD-10.

Since the patient continued to present as helpless and desperate and required a substantial amount of help and care by the nursing staff, admission to a residential home for patients with chronic neuropsychiatric diseases was discussed with the patient’s caregiver in light of the diagnosis of FTD and the lack of response to various medications. However, due to several encouraging case reports in the literature, we decided to attempt ECT with 10 sessions (instead of the usual 12 sessions) to minimize the risk of cognitive impairment [19, 20]. Bifrontal electrode placement and brief pulse ECT were applied two times per week for ten serial treatment sessions using a Thymatron IV device (Somatics, Lake Bluff, IL, USA). Anesthesia was performed with propofol, remifentanil, rocuronium, and sugammadex (the latter was used to antagonize the effects of rocuronium). Initially, stimulation intensity was 30 %, but ad to be increased during the course of ECT to 70 % due t insufficient seizures (motoric response shorter than 20 seconds), which required restimulation during ECT sessions. Hypertensive crises occurred repeatedly during ECT sessions, which complicated the procedure.

After the fourth ECT session the patient reported a considerable improvement in mood. Overall, the patient’s contact behavior appeared much more sorted and less clinging. Fears and general anxiety declined substantially during ECT treatment. Moreover, nihilistic ideation disappeared. The patient was able to organize her daily routine on the ward much more independently. After completion of the ECT series (10 sessions), the patient achieved a total score of 11 out of 63 points in the BDI-II, which almost corresponded to remission of depressive symptoms. However, a certain restlessness with repetitive actions could still be observed. Results in neurocognitive testing by different versions of MoCA remained stable over the course of the ECT treatment.

To document the success of the therapy, we performed another ^18^F-FDG PET/CT after two further maintenance sessions of ECT, four months after the first examination. The follow-up ^18^F-FDG PET/CT showed a frontally accentuated glucose hypometabolism, albeit with a clear improvement compared to the previous images (Figs. 2A-B). The Standardized Uptake Value (SUV) peak in the central nervous system was 7.0 before therapy and 9.7 thereafter. In the tomograms scaled to the maximum of the follow-up examination, the lower uptake before therapy could be seen virtually throughout the brain (cerebral cortex, basal ganglia, cerebellum). Statistical Parametric Mapping (SPM) comparison of the respective studies to the same control population showed a significantly more extended, contiguous cluster of suprathreshold voxels before therapy (*p < 0.001*) with significant hypometabolism (total of almost 50,000 voxels). In contrast, after therapy there were four non-contiguous, significantly smaller (> 1,000 voxels) clusters of 5,700, 7,000, 5,600 and 1,000 voxels (i.e., a total of approximately 19,000 voxels). In addition, a higher T_max_ value of 7.57 before therapy vs. 6.30 after therapy (peak height) was found as a significance maximum of hypometabolism.

We were finally able to discharge the patient back to her home environment after several months of stay at our university hospital. At home, the patient currently receives a low level of support from a nursing service. In our hospital, the patient continued regular ECT maintenance sessions for almost 6 months to maintain the success of the therapy, initially weekly. Over time the interval was gradually extended to 4 weeks.

## Discussion and conclusions

We reported the case of a 65-year-old female patient who displayed a severely depressed mood and concomitantly presented delusional, nihilistic thoughts. In the course of inpatient treatment, cognitive deficits were detected, which—in conjunction with the patient’s behavioral alterations—suggested the diagnosis of bv-FTD. To verify this, we performed an ^18^F-FDG brain PET/CT, which revealed profound glucose hypometabolism in frontal and parieto-temporal cortices. Since the patient did not respond to various antidepressant drugs, we performed ECT as an individual treatment trial. In the course of ECT, there was a significant improvement in the patient’s mood and depressive symptoms. Delusional experiences also disappeared. A follow-up ^18^F-FDG PET/CT showed persistent glucose hypometabolism, albeit with significant increase in metabolic activity compared with the previous examination.

The presented case illustrates the pitfalls in the clinical differentiation of depressive disorders from dementia [11]. Depressive episodes in older age have been discussed in the literature as precursors of dementia or as a reaction to an onset of dementia [11]. The phenomenon of depressive pseudodementia, which in contrast to classic dementia is amenable to antidepressant therapy, is also sometimes considered as a precursor of later dementia [21]. The patient’s clinical presentation with clinging contact behavior, formulation of diverse concerns and bizarre, repetitive psychomotor activity can be explained by disinhibition and stereotypical behaviors typical of bv-FTD [2]. A loss of empathy could also be verified in the patient. Apathy, which often presents in the initial phase of bv-FTD, and which needs to be distinguished from listlessness in the context of depression, was not present in our patient. On the contrary, the anxiety, rumination, and nihilistic symptoms could be explained by a psychotic depressive episode. Overall, we assumed a mixed picture of dementia and psychotic depression, although the question of coincidence or causal relationship between the two disorders could not be answered definitely.

Bv-FTD itself can only be treated symptomatically and with limited success with SSRIs, whereas curative treatment options exist for depression [2]. In the case of therapy resistance to classical antidepressants, as in our patient, ECT is often the therapy of choice. In studies, ECT has been shown to be superior to pharmacological therapies in terms of its antidepressant efficacy [22]. In older depressed patients with existing cognitive deficits or a diagnosis of dementia, the indication for ECT is often established rather cautiously, as further memory and concentration deficits are feared as a side effect of ECT. Studies have shown that ECT is particularly effective in older patients with depression and psychotic symptoms [23, 24]. Regarding the effectiveness of ECT in depressive symptoms and preexisting cognitive deficits, smaller studies showed that ECT treatment does not lead to further deterioration of memory deficits but causes an improvement in affective symptoms [25]. It should be noted that the case numbers of these studies were small and, therefore, the generalizability of these findings is very limited.

In addition, since there were some encouraging case reports regarding the improvement of depressive mood in comorbid dementia following treatment with ECT, we opted for such an approach in the presented case [19, 20]. We initiated a serial therapy with slightly reduced treatment frequency twice per week and bifrontal electrode placement to avoid cognitive side effects as much as possible [26]. We also performed only 10 serial therapies instead of the usual 12 sessions. Bifrontal electrode placement is associated with fewer side effects and similar effectiveness as compared to right unilateral or bitemporal variants [26]. Under this regime, there was an almost complete remission of depressive symptoms in the patient, which was also reflected by psychometric testing. Fortunately, no further cognitive impairment was registered during ECT. Likewise, delusional ideas disappeared. Regarding contact behavior, however, a certain disinhibition with stereotypies was still present, so that we ultimately assumed a coexistence of psychotic depression (which displayed a clear regression under ECT) and bv-FTD (which could not be treated curatively). It should be noted that 6 months after the end of the ECT series, an increase to 26 points was recorded in the BDI-II. However, due to further reduced subjective complaints of the patient, as well as the risk of further cognitive deterioration, it was decided against another ECT series.

The changes in brain metabolism and functional connectivity associated with depression, as well as their changes in the context of ECT, have been studied extensively [27]. For example, Mo et al. demonstrated by resting-state functional magnetic resonance imaging (rs-fMRI) that functional connectivity in the left angular gyrus improves in patients undergoing bifrontal ECT [28]. Likewise, an increase in connectivity under ECT between the dorsolateral prefrontal cortex and limbic regions was identified as a predictor of treatment response [29]. Taking changes in cerebral glucose metabolism into account, contradictory statements exist [30]. Commonly, depression as such is often associated with glucose hypometabolism in the prefrontal cortex [30]. Reininghaus et al. found no significant changes in brain metabolism during ECT, whereas other studies reported increases or decreases in cerebral glucose metabolism [30, 31]. Two different case reports also presented a complete normalization of brain metabolism in patients with depression under treatment with ECT [32, 33]. To the best of our knowledge, our report is the first in the literature to describe an improvement of glucose hypometabolism in different brain regions in a patient with bv-FTD and also alleviation of psychotic depression subsequent to ECT treatment. The fact that hypometabolism persisted even during maintenance therapy, together with the clinical picture, clearly indicates the diagnosis of bv-FTD, whereas the improvement of the depressive symptoms reflects the therapy response to ECT.

In summary, the presented case demonstrates the possibility of treating depressive symptoms associated with concurrent dementia with ECT when pharmacological therapy fails, although a single successful case does not allow for general recommendations. Bifrontal electrode placement and a lower frequency of ECT sessions might be beneficial in older patients. It should be noted that memory impairment as a side effect of ECT has been investigated only in small studies [34]. The positive outcome of the present study could be used, among other sources, as an impetus to develop general guidelines for the suitability of ECT in FTD. We were able to highlight the importance of functional imaging techniques, more specifically ^18^F-FDG PET/CT, for the differentiation of depression and dementia.

## Data Availability

All data generated or analyzed during this study are included in this published article.
